# Radiomic assessment of oesophageal adenocarcinoma: a critical review of 18F-FDG PET/CT, PET/MRI and CT

**DOI:** 10.1186/s13244-022-01245-0

**Published:** 2022-06-17

**Authors:** Robert J. O’Shea, Chris Rookyard, Sam Withey, Gary J. R. Cook, Sophia Tsoka, Vicky Goh

**Affiliations:** 1 Department of Cancer Imaging, School of Biomedical Engineering and Imaging Sciences, King’s College London, 5th floor, Becket House, 1 Lambeth Palace Rd, London, SE1 7EU UK; 2 Department of Radiology, The Royal Marsden NHS Foundation Trust, London, UK; 3 King’s College London & Guy’s and St Thomas’ PET Centre, St Thomas’ Hospital, London, UK; 4 Department of Informatics, School of Natural and Mathematical Sciences, King’s College London, London, UK; 5 Department of Radiology, Guy’s and St Thomas’ NHS Foundation Trust, London, UK

**Keywords:** Oesophageal neoplasms, Adenocarcinoma, Prognosis, Machine learning, Precision medicine

## Abstract

**Objectives:**

Radiomic models present an avenue to improve oesophageal adenocarcinoma assessment through quantitative medical image analysis. However, model selection is complicated by the abundance of available predictors and the uncertainty of their relevance and reproducibility. This analysis reviews recent research to facilitate precedent-based model selection for prospective validation studies.

**Methods:**

This analysis reviews research on 18F-FDG PET/CT, PET/MRI and CT radiomics in oesophageal adenocarcinoma between 2016 and 2021. Model design, testing and reporting are evaluated according to the Transparent Reporting of a Multivariable Prediction Model for Individual Prognosis or Diagnosis (TRIPOD) score and Radiomics Quality Score (RQS). Key results and limitations are analysed to identify opportunities for future research in the area.

**Results:**

Radiomic models of stage and therapeutic response demonstrated discriminative capacity, though clinical applications require greater sensitivity. Although radiomic models predict survival within institutions, generalisability is limited. Few radiomic features have been recommended independently by multiple studies.

**Conclusions:**

Future research must prioritise prospective validation of previously proposed models to further clinical translation.

**Supplementary Information:**

The online version contains supplementary material available at 10.1186/s13244-022-01245-0.

## Key points

- Radiomic predictor recommendations vary considerably between studies.
- Although radiomic models have demonstrated discriminative predictions in oesophageal cancer tasks, adequate sensitivity has yet to be demonstrated.
- Future radiomic research in oesophageal adenocarcinoma should prioritise validation of previously proposed predictors over further feature selection.

## Background

Oesophageal adenocarcinoma presents a major disease burden worldwide, with age-standardised incidence of 0.9 per 100,000 and 1-year survival of 47–55% [1, 2]. Although therapeutic developments have improved survival [2, 3], scope remains to optimise management through improved staging, therapeutic response prediction and prognostication [4–6]. Radiomics—the analysis of quantitative medical imaging features describing morphology, texture and intensity distribution—is a non-invasive method to assess oesophageal adenocarcinomas through quantification of tumour characteristics.

The search for optimal radiomic models is complicated by the breadth of candidate radiomic features and learning algorithms, which present an enormous parameter space to screen. Sample sizes are limited in clinical imaging studies, creating a scenario in which data-driven feature selection can be unreliable [7–9]. The variation of radiomic feature distributions with imaging equipment, acquisition parameters and annotation methodology presents an additional obstacle for model generalisation [5, 9–11]. Methodological rigour is essential to control false detection rates in such conditions [9], and several reviews have raised concerns regarding design and reporting of imaging models [11–14]. Unsurprisingly, 76% of proposed radiomic predictors are estimated to be false positives [14].

To alleviate biases associated with model selection in individual data sets, studies may validate previously proposed features and models. This approach is a necessary development in the transition from exploration to testing, carrying an appropriate weight in the Radiomics Quality Score (RQS) [15]. However, the complexity of radiomic feature definitions and nomenclature complicate aggregation of results from different studies, hampering validation reproducibility. Accordingly, recent initiatives are now being made in an attempt to standardise radiomic features [15]. This review inspects and evaluates radiomic analyses focussing on the oesophageal adenocarcinoma subtype from a methodological standpoint, extracting features under a unified nomenclature to facilitate future validation studies. The exploratory phase of oesophageal cancer radiomics was well characterised in Van Rossum’s 2016 review [16], and here we review subsequent research and developments.

## Materials and methods

A literature search was performed to identify original research articles applying radiomics or artificial intelligence to predict stage, therapeutic response or prognosis in human oesophageal adenocarcinoma using PET/CT, PET/MRI or CT images. Searches were conducted on Embase and MEDLINE databases for full-text articles published in peer-reviewed journals in the English language between 1 January 2016 and 4 January 2022. Search queries are provided in Additional file 1. References of included studies were also screened. Studies with fewer than 10 adenocarcinoma cases, those with squamous cell carcinoma only, and those which omitted histological information were excluded. This threshold reflected the recommended minimum sample size for univariate cox model training [17, 18], whilst avoiding the exclusion of studies with low sample sizes but high quality, such as prospective validation analyses. Where histology-specific results were unavailable, aggregate results were extracted. Studies which modelled both oesophageal and gastroesophageal junction adenocarcinomas were included in this analysis. Transparent Reporting of a Multivariable Prediction Model for Individual Prognosis or Diagnosis (TRIPOD) score [19] and RQS [15] were annotated where applicable. Model validation was classified as “internal”, “temporal” or “external” according to whether the data partition represented (1) a random split, (2) a split after a specific time point or (3) a different institution. Radiomic features were extracted and annotated according to Image Biomarker Standardization Initiative nomenclature [20] in the format “Family_Feature”. A maximum of five features were extracted from each study, according to the most significant associations or model contributions. Radiomic feature selection frequency was estimated for studies analysing primary tumoural radiomics. Visualisation was performed with R, RStudio and ggplot [21–23]. Discrimination performance (e.g. how appropriately a predictor ranks patients with respect to 1-year survival) was quantified by area under the receiver operating characteristic curve (AUC). Categorical associations (e.g. survival time differences between participant groups) were described with ${\chi }^{2}$ metrics. Continuous associations (e.g. association of a radiomic feature with volume change) were described with Pearson’s correlation ($\rho )$. Cox regression model coefficients (which quantify predictors’ contributions a prognostic model) were described by the hazard ratio (HR). Clinical tumour, node and metastasis stages were abbreviated as cT, cN, cM and cTNM; and corresponding post-neoadjuvant pathological stages as ypT, ypN, ypM and ypTNM.

## Results

**Fig. 1:**
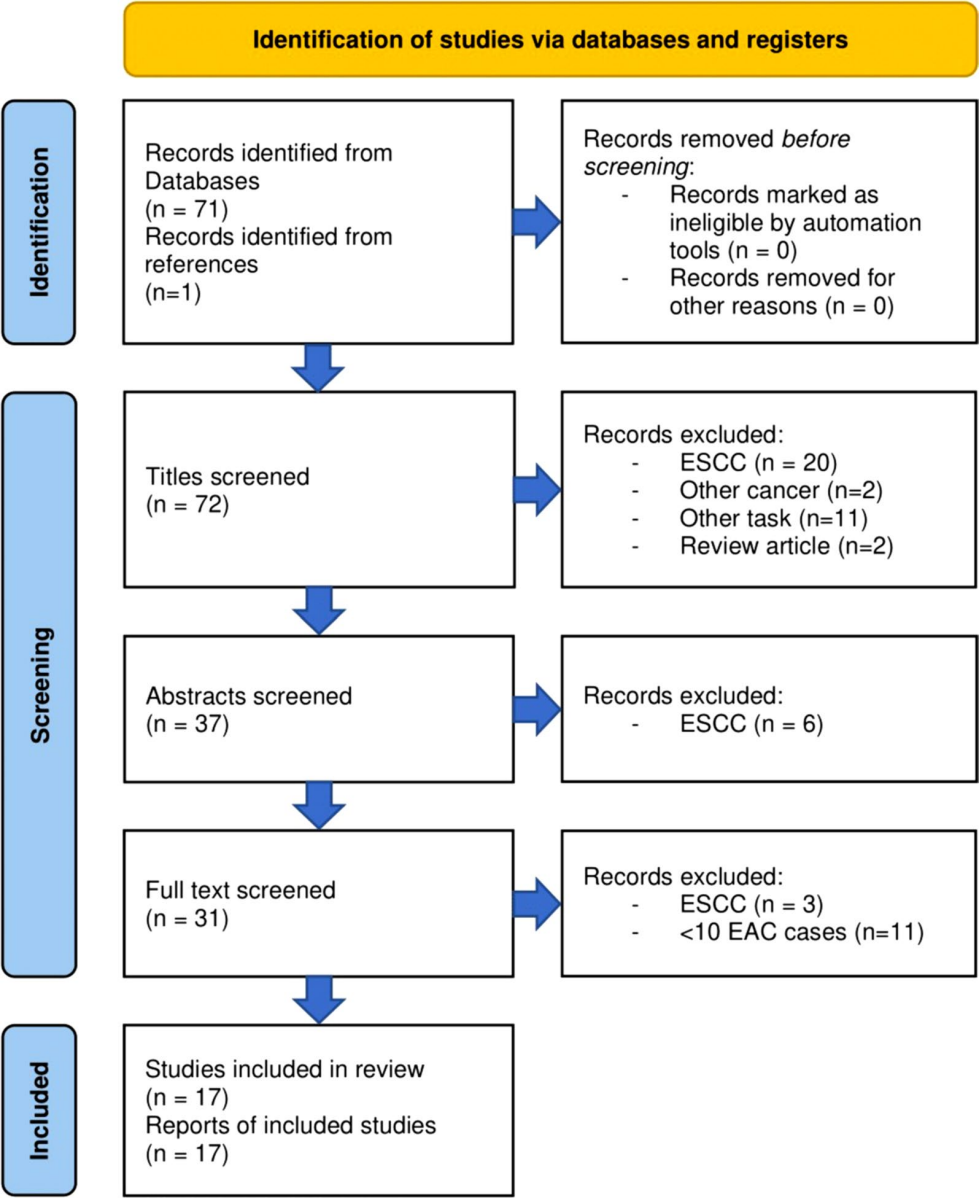
Flow chart of article screening and inclusion. *ESCC* oesophageal squamous cell carcinoma, *EAC* oesophageal adenocarcinoma

**Fig. 2:**
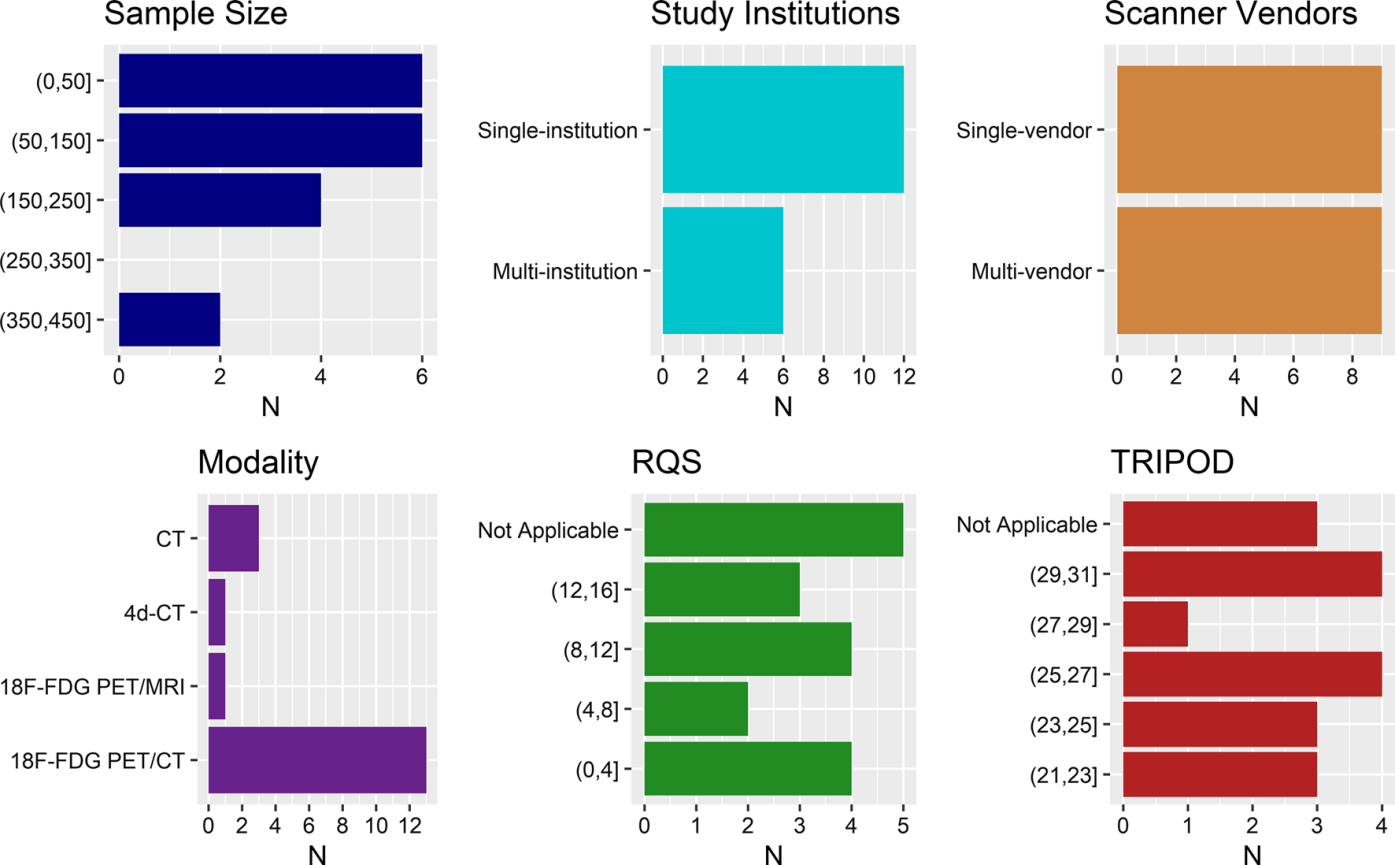
Histograms of information on included articles. Upper left: study sample size. Upper middle: number of institutions from which data were collected. Upper right: number of scanner vendors with which images were acquired. Lower left: image modality. Lower middle: Radiomics Quality Score (RQS). Lower right: Transparent Reporting of a Multivariable Prediction Model for Individual Prognosis or Diagnosis (TRIPOD) score

Articles (*n* = 72) were screened and 17 were included in this analysis. A flow diagram of the screening process is provided in Fig. 1 [24]. Article information is summarised in Fig. 2. Results and predictive features from the five studies with the highest RQS are provided in Table 1. Article screening is detailed in Additional file 1: Data S1. TRIPOD annotations are provided in Additional file 1: Data S2. RQS annotations are provided in Additional file 1: Data S3.

**Table 1:** Results and predictive features in the seven studies with the highest RQS and TRIPOD score

Study	Scores	Modality	*N*	Task	Performance	Radiomic features
[6]	*RQS:* 16 *TRIPOD:* 27	18F-FDG PET	73	Response (TRG = 1)	*Internal:* AUC: 0.81	1. GLCM_AngularSecondMoment
[27]	*RQS:* 14 *TRIPOD:* 26	18F-FDG PET	96	Response (TRG = 1)	*Internal:* AUC: 0.82	1. Shape_GearysCMeasure 2. GLRLM_LongRunLowGrey LevelEmphasis
[10]	*RQS:* 13 *TRIPOD:* 31	18F-FDG PET	403	OS	*Internal:* ${\chi }_{3}^{2}$: 143.14 *p* < 0.001	1. IntensityHistogram_Energy 2. IntensityHistogram_Kurtosis
[43]	*RQS:* 25 *TRIPOD:* 31	18F-FDG PET	46	OS	*External:* ${\chi }_{3}^{2}$: 1.27 *p* = 0.74	1. IntensityHistogram_Energy 2. IntensityHistogram_Kurtosis
[25]	*RQS:* 17 *TRIPOD:* 29	18F-FDG PET	190	ypN stage	*Internal:* AUC: 0.82 95% CI [0.74–0.89] *External:* AUC: 0.69 95% CI [0.54–0.8]	1. NGTDM_DependenceEntropy 2. Shape_VolumeDensity 3. NGTDM_Coarseness 4. IntensityHistogram_Minimum HistogramGradient 5. GLCM_InverseDifference MomentNormalised
[25]	,,	,,	,,	OS	*External:* ${\chi }_{3}^{2}$: 6.08 *p* = 0.01	,,
[41]	*RQS:* 12 *TRIPOD:* 24	CT	239	OS (3 yr)	*Internal:* AUC: 0.69 95% CI [0.61–0.77] *External:* AUC: 0.61 95% CI [0.47–0.75]	1. GLCM_InverseVariance 2. GLDZM_LowGreyLevelZone Emphasis 3. GLRLM_RunLengthNon Uniformity 4. GLCM_InformationMeasure OfCorrelation1 5. NGLDM_DependenceCount Nonuniformity
[4]	*RQS*: 12 *TRIPOD*: 31	18F-FDG PET	217	Response (TRG = 1)	*Internal:* AUC: 0.77 95% CI [0.70–0.83]	1. GLCM_ClusterShade 2. GLRLM_RunPercentage 3. GLCM_JointEntropy 4. Shape_Sphericity

*AUC* area under receiver operator characteristic, *RQS* Radiomics Quality Score, *TRIPOD* Transparent Reporting of a Multivariable Prediction Model for Individual Prognosis or Diagnosis, *TRG* tumour regression grade, *OS* overall survival, $y$*pN* post-neoadjuvant nodal status, *GLCM* grey-level co-occurrence matrix, *GLRLM* grey-level run length matrix, *NGTDM* neighbouring grey tone difference matrix, *NGLDM* neighbouring grey-level dependence matrix

### Staging

#### 18F-FDG PET

Two 18F-FDG PET studies modelled stage. Zhang modelled ypN on retrospective 18F-FDG PET/CT data from patients receiving chemoradiotherapy (CRT) in two institutions (TRIPOD: 29, RQS: 17) [25]. All 190 patients had adenocarcinoma and underwent neoadjuvant chemoradiotherapy prior to surgery. Supervised feature elimination and L1-penalisation selected four clinical features (age, clinical t-stage (cT), treatment, tumour regression grade (TRG)) and nine radiomic features. PET radiomics added minimal information to clinical features in internal validation (AUC: 0.82 vs. 0.79, *p* = NR), and the models were equivalent in external validation (AUC: 0.69 vs. 0.65, *p* > 0.05). In external validation, cN demonstrated similar discrimination but greater sensitivity, compared with clinical (AUC: 0.66 vs. 0.69, sensitivity: 0.89 vs. 0.52) and clinicoradiomic models (AUC: 0.66 vs. 0.65, sensitivity: 0.89 vs. 0.63).

Baiocco modelled baseline metastatic status retrospectively on prospectively collected serial 18F-FDG PET/MRI data from a single institution (TRIPOD: 24, RQS: 4) [26]. Seventeen out of 20 participants had adenocarcinoma. In training data, a bivariate model of gross tumour volume radiomics (SUV grey-level co-occurrence matrix (GLCM) GLCM_JointEntropy, ADC GLCM_JointEntropy) demonstrated moderate discrimination (accuracy 0.8, *p* < 0.001). Adjustments for multiple hypothesis testing were not performed in this exploratory study.

## Therapeutic response

### Summary of studies

#### 18F-FDG PET

Seven studies modelled therapeutic response. Beukinga modelled CRT response on retrospective serial 18F-FDG PET data from patients with locally advanced disease at a single institution (TRIPOD: 27, RQS: 16) [6]. Sixty-three out of 73 patients had adenocarcinoma. Baseline and neoadjuvant radiomic features were evaluated for robustness to segmentation by different annotators. Twelve models were developed with various combinations of clinical and radiomic features. In internal validation, a clinicoradiomic model (cT, post-therapeutic GLCM_AngularSecondMoment) discriminated complete response (TRG = 1) better than clinical features (cT, histology) alone (AUC: 0.81 vs. 0.75, *p* = NR).

In a separate cohort, Beukinga modelled neoadjuvant CRT response on retrospective 18F-FDG PET and genomic data from patients with locally advanced disease at a single institution (TRIPOD: 26, RQS: 14) [27]. Eighty-eight out of 96 patients had adenocarcinoma. Hierarchical clustering was employed to select clinical (cT, histology) and radiomic features (Shape_GearysCMeasure, grey-level run length matrix (GLRLM) GLRLM_LongRunLowGreyLevelEmphasis). In internal validation, incorporation of gene amplification data (cluster of differentiation 44 and human epidermal growth factor receptor 2 genes) improved clinicoradiomic discrimination of complete response (TRG = 1) (AUC: 0.82 vs. 0.69, *p* = NR). Weaker performance was achieved with clinical features alone (AUC: 0.82 vs. 0.66, *p* = NR).

Van Rossum modelled CRT response retrospectively on serial 18F-FDG PET data from oesophageal cancer patients at a single institution (TRIPOD: 31, RQS: 12) [4]. Forty-four out of 45 patients had adenocarcinoma. Radiomic feature stability was quantified in a subcohort of 7 patients who had baseline imaging repeated in two institutions. Logistic regression models were generated from clinical (tumour length, cT, therapy, tumour, residual disease on post-CRT biopsy, regression grade) and radiomic features (baseline GLCM_ClusterShade, post-therapeutic metabolic_TumourLesionGlycolysis, post-therapeutic Shape_Sphericity, delta GLRLM_RunPercentage, delta GLCM_JointEntropy). In internal validation, radiomic features improved discrimination of complete (TRG = 1) response (AUC: 0.77 vs. 0.72, *p* = NR). Radiomics were not found to add value at a sensitivity threshold (90%) which could select patients to forego surgery. As the validation set was employed for model optimisation, performance may have been overestimated.

Yip modelled CRT response on retrospective serial 18F-FDG PET data from oesophageal cancer patients at a single institution (TRIPOD: 23, RQS: 3) [28]. Fifty out of 54 patients had adenocarcinoma. Six radiomic features (GLCM_Homogeneity, GLCM_JointEntropy, GLRLM_HighGreyLevelRunEmphasis, GLRLM_ShortRunHighGrayRunEmphasis, grey-level size zone matrix (GLSZM) GLSZM_HighGrayLevelZoneEmphasis, GLSZM_SmallZoneHighGreyLevelEmphasis) were preselected based on previous studies, and deltas were evaluated. In training data, delta GLCM_JointEntropy discriminated partial response (ypTNM < cTNM) from non-response (AUC: 0.71, *p* = 0.01). However, complete response (ypT = 0) was not distinguished. Partitioned model validation was omitted.

Simoni modelled CRT response retrospectively on a prospective 18F-FDG PET data from patients with locally advanced disease at a single institution (TRIPOD: 23, RQS: 3) [29]. Thirty-five out of 53 patients had adenocarcinoma. Radiomic dimensionality reduction was performed with unsupervised clustering, and five representative features were considered. In training data, two radiomic features (baseline GLCM_JointEntropy and baseline GLCM_InverseDifferenceNormalised) demonstrated univariate associations with response (TRG $\le$ 2).

#### CT

Zhang modelled CRT response retrospectively using PET segmentation to support CT radiomic extraction from serial 18F-FDG PET/CT data from a single institution (TRIPOD: 30, RQS: 5) [30]. Although data were sourced from a multicentre trial, patients with images recorded in other institutions were excluded. One hundred fifty-four out of 181 patients (84%) were excluded in total. Nineteen out of 29 included patients had adenocarcinoma. Five radiomic features were preselected based on previous studies. Although adjustment for multiple hypothesis testing was not reported, deltas in three radiomic features (GLCM_InverseDifferenceMoment, GLCM_Contrast, GLCM_Correlation) would have remained significantly associated with response (ypT $\le 2$) under Bonferroni correction. Survival associations (OS $\ge$ 1 yr) were not identified.

Klaasen modelled chemotherapy response on retrospective serial CT data from stage IVb patients from multiple institutions, extracting radiomic features from hepatic metastases (TRIPOD: 31, RQS: 10) [31]. One hundred ninety-six lesions were included in the analysis. Sixteen out of 18 patients had adenocarcinoma. Patients were restricted to those with visible liver metastases on baseline and post-therapeutic scans. The random forest algorithm was applied to model 370 radiomic features, extracting feature importance according to Gini index. In patient disjoint internal validation, a radiomic model discriminated complete (no residual tumour on second scan) response (AUC: 0.79 [0.74–0.88]). Partial response (> 65% volume reduction) was not discriminated as easily (AUC: 0.64 [0.55–0.73]). It is noted that radiomic distributions may differ between primary and metastatic lesions, as was observed by Wagner in a cohort of patients with metastatic colorectal cancer [32].

### Outcome discretisation

Where studies discretised continuous variables, valuable information may have been lost [33]. Although TRG is predictive of OS [34], Zhang found no correlation between dichotomised survival (OS > 1 yr) and predictors of dichotomised response (ypT $\le 2$) [30]. Klaassen dichotomised partial response at 65% volume reduction, according to a computational measurement [31]. Consequently, trivial clinical differences between 64 and 66% tumour volume reduction may have been overrepresented, whilst significant differences between 0 and 64% underrepresented. Indeed, this model learned highly nonlinear decision surfaces—the second most important feature (GLCM_ClusterShade) was perfectly uncorrelated with actual volume decrease (Gini Index: 1.44, Pearson *r*: 0.0). Furthermore, less important features such as GLCM_InformationMeasureOfCorrelation1, which correlated strongly with volume decrease (Gini Index: 0.81, Pearson *r*: 0.55) would have yielded informative linear predictors.

### Selection bias in therapeutic response studies

Selection biases were apparent in several studies. For example, Beukinga, Zhang and Van Rossum excluded participants with images recorded in other institutions, potentially reducing model generalisability [4, 6, 30]. Klaasen restricted their cohort to patients with visible hepatic metastases on both baseline and post-therapeutic scans [31]. Consequently, the model only observed complete lesion regression in the presence of other visible disease. The generalisability of these findings to the clinically preferable outcome in which all lesions regress cannot be guaranteed.

### Feature preselection

Feature preselection avoids severe adjustments for multiple hypothesis testing, thereby optimising statistical power to detect relevant features within the preselected set. Accordingly, Yip and Foley considered a limited number of radiomic features suggested by previously published results [10, 35–39]. Piazzese and Van Rossum quantified feature stability in subcohorts with images recorded in separate institutions, preselecting features with stable distributions a priori [5, 10]. Although Klaassen preselected feature families based on a previous analysis [40], 370 variables were included in the analysis [31]. Beukinga and Baiocco performed unsupervised feature selection by clustering, conserving power to test a small number of selected variables against the response [26, 27]. Larue and Zhang employed supervised feature selection [25, 41]—this approach retains overfitting risks as the response is observed. Both analyses provided unbiased estimates of model performance through external validation—out-of-sample performance decreases demonstrated overfitting in each case.

### Clinical applicability

To inform surgical management decisions, therapeutic response models must demonstrate sensitivity to residual disease, i.e. if watch-and-wait is to be considered following neoadjuvant therapy, models must provide high certainty of complete response. Metrics such as AUC and accuracy may misrepresent performance in this regard. Van Rossum and Yip both identified poor sensitivity to residual disease, highlighting the importance of clinically focussed modelling objectives [4, 28]. Most radiomic models will provide imperfect information—i.e. they improve risk predictions somewhat, but retain relatively high error rates. Such models may be applied more securely in scenarios where the risk–benefit ratios are uncertain, such as in the selection between two therapeutic approaches with similar efficacies. Other potentially valuable applications include therapeutic dose optimisation, as was demonstrated by Her in the optimisation of intensity-modulated radiotherapy for prostate cancer [42].

## Survival

### Summary of studies

#### 18F-FDG PET

Six studies modelled overall survival (OS). Foley modelled OS on retrospective 18F-FDG PET data from a single institution (TRIPOD: 31, RQS: 13) [10]. Out of 403 participants, 316 had adenocarcinoma. Backwards conditioning was employed to select three clinical features (age, cTNM and treatment intent) and three radiomic features (metabolic_TumourLesionGlycolysis, IntensityHistogram_Energy and IntensityHistogram_Kurtosis) from 19 preselected features. In temporally partitioned validation, clinicoradiomic model quartiles contained more survival information than clinical model quartiles (${\chi }_{3}^{2}$: 143.1 vs. 20.6, *p* = NR).

In a separate study, Foley validated their proposed model on prospective 18F-FDG PET data from three institutions (TRIPOD: 31, RQS: 25) [43]. Thirty-nine out of 46 participants had adenocarcinoma. Neither clinicoradiomic model quartiles nor clinical model quartiles were found to associate with overall survival (${\chi }_{3}^{2}$: 1.4 vs. 1.2, *p* = NR). However, calibration slopes did not differ from unity, supporting preservation of discriminative capacity. Feature harmonisation was also performed with the “combat” algorithm [44], though performance remained similar.

Karahan modelled OS on retrospective 18F-FDG PET data from a single institution (TRIPOD: 23, RQS: 7) [45]. Thirteen out of 62 patients had adenocarcinoma histology. Forty-seven radiomic features were considered. Although several univariate associations were identified between radiomic features and survival outcomes, adjustments for multiple hypothesis testing were not reported—consequently, significance may have been overestimated. Nonetheless, in internal validation, logistic regression models demonstrated good discrimination of 1-year OS (AUC: 0.635) and 5-year OS (AUC: 0.82). Model features were not reported. Analysis was restricted to patients who were known to be alive or deceased at each time interval, resulting in the exclusion of 5/75 patients (7%) lost to follow-up in the first year and 15/75 (20%) lost in 5 years.

Zhang modelled OS in an external cohort using their 18F-FDG PET staging models (TRIPOD: 29, RQS: 17) [25]. The clinicoradiomic model predicted overall survival in the external data (${\chi }_{1}^{2}:6.08, P=0.01$).

#### CT

Piazzese modelled OS retrospectively on CT data from a multicentre randomised controlled trial (TRIPOD: 27, RQS: 4) [5]. Fifty-three out of 213 participants had adenocarcinoma, while the majority had squamous cell carcinoma. Radiomic stability was estimated by comparing feature distributions in 2D and 3D images. In a Cox regression model with five clinical features (age, sex, cTNM, WHO performance status, and IV contrast administration) and four stable radiomic features (GLCM_InverseVariance, grey-level distance zone matrix (GLDZM) GLDZM_LargeDistanceEmphasis, GLDZM_ZoneDistanceNonUniformityNormalised and GLDZM_ZoneDistanceVariance), GLDZM_ZoneDistanceVariance demonstrated significant association with survival (hazard ratio 1.25, *p* = 0.03). Omission of model validation was justified by prioritisation of false positive and negative rates in predictor selection—all observations were used for model fitting.

Larue modelled 3-year OS on retrospective CT data from two institutions (TRIPOD: 24, RQS: 12) [41]. Out of 239 participants, 193 had adenocarcinoma. Recursive feature elimination was employed to select 40 predictors from a set of 1049 radiomic features. The random forest algorithm was employed to model radiomic features (not reported) and clinical features (age, gender, histology, cTNM). Although radiomics outperformed clinical features in internal validation (AUC: 0.69 vs. 0.63, *p* = NR), similar performance was demonstrated in external validation (AUC: 0.61 vs. 0.62). Supervised feature selection and modelling were performed in separate runs of cross validation, rather than within cross-validation splits. This procedural error is common in radiomic analyses and consequent data leakage results in a bias towards overly complex models [13]. Indeed, decreased external validation performance indicated overfitting.

### Selection bias in survival studies

The retrospective time frame of survival analyses may result in various selection biases. Karahan performed two separate exclusions, removing those lost to follow-up at 1 year and 5 years, respectively. Consequently, participants lost to follow-up due to death were excluded from survival outcomes, inducing bias [46]. Larue excluded cases which did not undergo surgery, although this information would not be available for the immediate application of a pre-treatment imaging model [41]. Piazzese utilised trial data, and exclusions were not reported [5]. Foley’s exclusions were most suited to clinical application, as they were clearly described and based on contemporaneous variables with research precedent (SUV_max_ < 3, MTV < 5 ml, histology other than adenocarcinoma or squamous cell carcinoma, synchronous malignancies and oesophageal stenting) [10, 43]. Foley’s validation study provided the most unbiased estimates of model performance, as the model and exclusions were fixed prior to application in a prospective data set [43].

### Clinical applicability

As the clinical consequences of false positives and false negatives rarely equate, traditional model metrics may have limited relevance at patient level and further decision curve analysis may be required [4]. Larue found that their model demonstrated a 24% false negative rate for 3-year mortality, concluding that the model cannot support treatment decisions [41]. Beyond use for management decisions, radiomic survival models may find an important application in patient information. Oncology patients rank life expectancy as their highest information priority [47]. Consequently, an additional objective risk measure may improve patient-centred care if used appropriately. However, radiomic models’ complexity and reliability may prove difficult to communicate in practice.

## Evaluation of technical aspects

### Stability of radiomic features

#### 18F-FDG PET

Whybra [48] assessed radiomic feature robustness to resampling on retrospective 18F-FDG PET data from patients at a single institution (RQS: 3). Nineteen out of 131 patients had adenocarcinoma. Radiomic distributions were found to vary with interpolation method.

Van Rossum et al. [4] evaluated feature stability in a subset of their cohort with baseline 18F-FDG PET images recorded in both the institutions. Both segmentations were performed by a single clinician. Shape and metabolic features demonstrated high stability; first-order, GLCM and GLRLM features demonstrated moderate stability; and neighbouring grey tone distance matrix (NGTDM) features demonstrated poor stability.

#### CT

Larue [40] evaluated stability of radiomic features with respect to respiratory phase in 4D-CT data from patients at a single institution. Twenty out of 40 had adenocarcinoma. Wavelet filtered image features were found to be less robust than features computed on the original image. Shape features and GLDZM features were the most stable feature families overall.

### Impact of segmentation methods

Parkinson [49] evaluated the impact of segmentation methodology on survival models developed in Foley’s cohort [10] (TRIPOD: 27, RQS: 11). Six segmentation algorithms were applied. Radiomic features varied to the extent that some survival associations reversed.

Yip [35] modelled therapeutic response in retrospective serial 18F-FDG PET data from a single centre, evaluating the impact of contour propagation methodology (RQS: 3). Forty-four out of 45 patients had adenocarcinoma histology. Three preselected features’ deltas (GLCM_JointEntropy, GLRLM_ShortRunHighGreyRunEmphasis, GLZSM_ShortZoneHighGreyLevelEmphasis) were found to be robust to registration algorithm variation.

### Radiomic features’ volume dependence

Several studies identified associations between observed radiomic features and tumour volume [35, 49]. Following Hatt’s recommendations [50], Van Rossum and Foley excluded small tumours from their analyses [4, 25]. Volume confounding may be evaluated by inclusion of volume as a predictor [50]. Several studies also noted the limitation that radiomic features may vary according to segmentation method [10, 28, 31, 45].

### Radiomic feature selection frequency

**Fig. 3:**
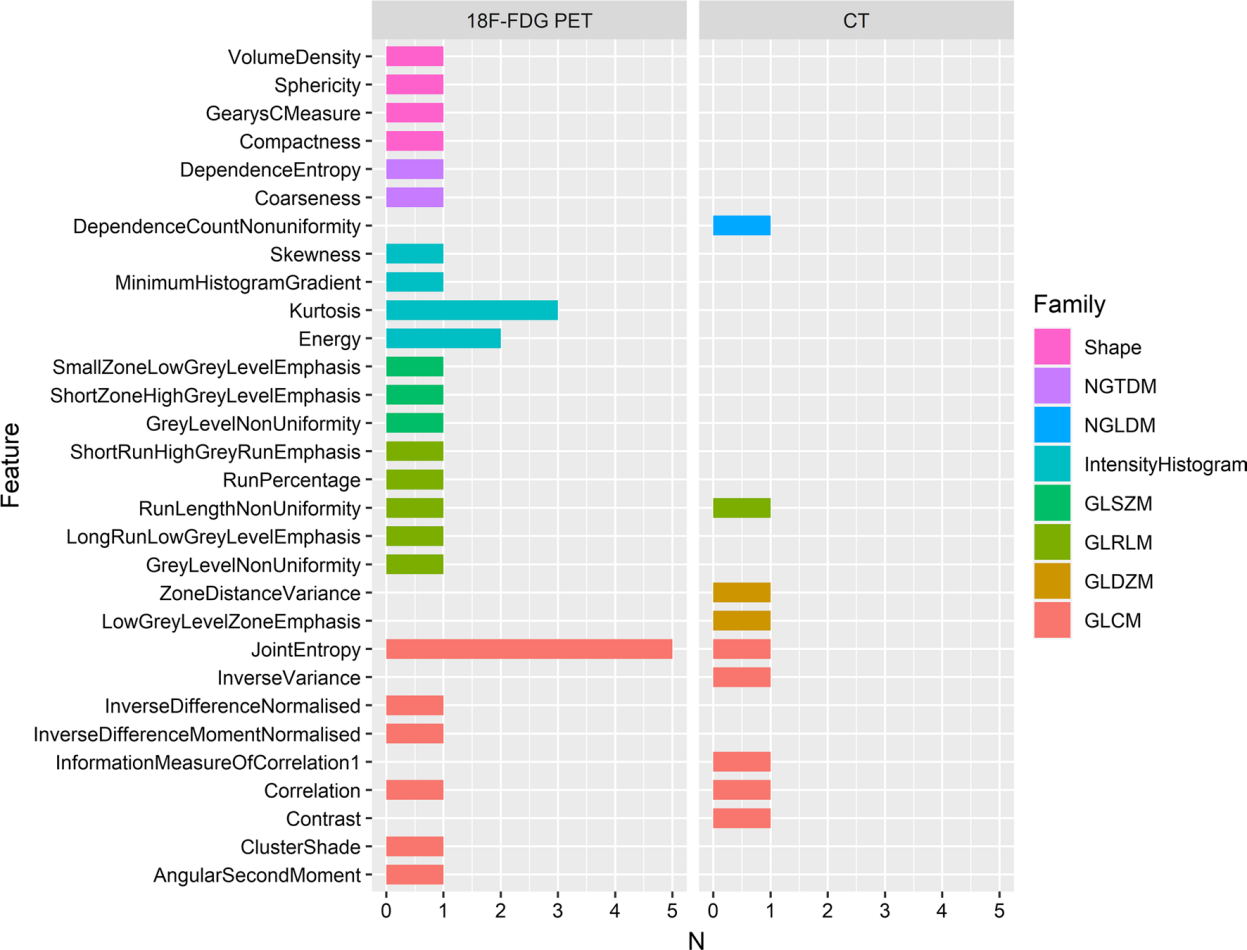
Histogram of radiomic feature recommendations by modality, excluding image transforms. Up to five features were extracted from each study, according to significance or model contribution. *GLCM* grey-level co-occurrence matrix, *GLDZM* grey-level distance-zone matrix, *GLRLM* grey-level run length matrix, *GLSZM* grey-level size zone matrix, *NGTDM* neighbouring grey tone difference matrix, *NGLDM* neighbouring grey-level dependence matrix

The feature space of radiomic models varied considerably between studies. Disregarding image transformations, 21/25 identified features were recommended by one study each. The most frequently selected feature was GLCM_JointEntropy, appearing in five PET studies [4, 28–30, 35] and one CT study [30]. A histogram of radiomic feature selection frequency is provided in Fig. 3. Significant radiomic features extracted from each article are provided in Additional file 1: Data S4.

## Discussion

### Summary of findings

Although staging models demonstrated discriminative capacity [25, 26], sensitivity afforded by radiologists was unmatched. Radiomic models of therapeutic response demonstrated marginally higher discrimination than clinical models in three studies [4, 6, 27]. However, radiomic features did not improve clinical models’ sensitivity to residual disease where evaluated [4, 28]. Survival models were informative; however, generalisability was limited [5, 10, 41, 43, 45].

### Design and reporting standards

Many studies followed design and reporting recommendations—Foley followed Moons’ biomarker development recommendations [10, 43, 51] and Klaasen, Larue, Van Rossum, Piazzese and Zhang cited TRIPOD guidance [4, 5, 25, 41]. TRIPOD scores ranged from 23/31 to 31/31, indicating comprehensive reporting. TRIPOD compliance enhanced transparency and reproducibility. However, RQS ranged from 3/36 to 25/36 with a median score of 11/36, highlighting many opportunities for design improvement from a radiomics perspective. In particular, only one prospective validation study was identified [43]. These findings concur with previous analyses [11, 12, 52, 53].

### Modelling algorithms

Minimally complex models such as logistic regression, linear discriminant analysis and cox regression were employed in most studies [4–6, 10, 25, 27, 30, 45, 49]. The small parameter spaces of these algorithms suited the limited sample sizes available, particularly where full sets of radiomic predictors were considered. Regression-based models are also amenable to biological interpretation as coefficients describe the direction and magnitude of the estimated effects. Klaasen and Larue [31, 41] developed random forest models. The instability of importance measures in the high-dimensional setting [54] complicates application of random forest modelling to radiomic feature selection—this approach is best applied to data sets where observations outnumber variables [9]. Indeed, Larue’s model demonstrated decreased performance in external validation [41]. Furthermore, random forest decision functions require complex descriptions, impending reproduction.

### External validation and generalisability

Radiomic feature distributions may differ between centres, due to variability in scanner model, acquisition parameters and population characteristics [6, 10, 25, 27, 31, 45], prompting calls for standardisation of these parameters [27, 41, 45]. Accordingly, Piazzese, Beukinga and Karahan preselected features on the basis of stability [5, 6, 45]. Both studies testing inter-institutional generalisability demonstrated performance decreases [25, 41], indicating that some degree of overfitting occurred. Lack of external validation was frequently cited as a limitation [6, 25, 27, 29–31, 41]. Of five studies which performed internal validation, only Foley reported the performance of a single finalised model on test data which was unobserved during training or model selection [10]. Although Foley did not find their model performance significant in external validation, it should be noted that the small sample size of the external data set limited the power to detect significant results [43].

### Study limitations

Recognising the distinct clinical prognostic profiles of oesophageal adenocarcinoma and squamous cell carcinoma [2], the studies analysed in this review were selected to provide a large predominance of adenocarcinomas. However, the inability to completely separate the small amount of squamous cell carcinoma data in some included studies may reduce specificity of the feature recommendations for a pure adenocarcinoma cohort. Comparison of individual study findings was also complicated by variability in the considered features, selection methods and modelling algorithms. Furthermore, statistical measures of association varied, precluding conventional meta-analysis. Comparative evaluation of image modalities was further precluded due to the paucity of studies evaluating CT and MRI. Our study reports frequency of feature selection, ignoring significance and direction of effect in individual studies. Inclusion of small studies allowed for faithful representation of the diverse conditions across different studies—however, greater feature selection variability and lower feature significance may be expected in smaller studies. Finally, this analysis was limited to studies published in the English language.

## Conclusions

Radiomic models for 18F-FDG PET, MRI and CT have been proposed for staging, therapeutic response assessment and prognostication. Many studies have reported significant results. An urgent clinical need exists for a generalisable, rigorously tested prognostic model for oesophageal adenocarcinoma. Thus, future studies must prioritise unbiased model validation over further exploratory research. This review consolidates study findings and proposes features to facilitate precedent-based design of prospective radiomic studies.

## Supplementary Information

**Additional file 1: S1.** Details of all screened articles.**S2.** Tripod scores of included articles.**S3.** Radiomic quality scores of screened articles. **S4.** Significant radiomic features in included articles.

## Data Availability

All studies included in this analysis are available from their relevant publications. Search queries are provided in Additional file 1. Article screening is detailed in Additional file 1: Data S1. TRIPOD annotations are provided in Additional file 1: Data S2. RQS annotations are provided in Additional file 1: Data S3. Significant radiomic features extracted from each article are provided in Additional file 1: Data S4.

## Abbreviations

AUC: Area under receiver operator characteristic
CRT: Chemoradiotherapy
cTNM, cT, cN, cM: Clinical tumour node metastasis stage
GLCM: Grey-level co-occurrence matrix
GLRLM: Grey-level run length matrix
GLSZM: Grey-level size zone matrix
HR: Hazard ratio
NGLDM: Neighbouring grey-level dependence matrix
NGTDM: Neighbouring grey tone difference matrix
NR: Not reported
OS: Overall survival
PFS: Progression-free survival
RQS: Radiomics Quality Score
TRG: Tumour regression grade
TRIPOD: Transparent Reporting of a Multivariable Prediction Model for Individual Prognosis or Diagnosis
ypTNM, ypT, ypN, ypM: Post-neoadjuvant pathological tumour node metastasis stage
$\rho$: Pearson’s correlation coefficient
${\chi }_{k}^{2}$: Chi-squared statistic with $k$ degrees of freedom

## References

[1] Arnold M, Ferlay J, Van Berge Henegouwen MI, Soerjomataram I (2020). Global burden of oesophageal and gastric cancer by histology and subsite in 2018. Gut.

[2] Morgan E, Soerjomataram I, Gavin AT (2021). International trends in oesophageal cancer survival by histological subtype between 1995 and 2014. Gut.

[3] Shapiro J, van Lanschot JJB, Hulshof MCCM (2015). Neoadjuvant chemoradiotherapy plus surgery versus surgery alone for oesophageal or junctional cancer (CROSS): long-term results of a randomised controlled trial. Lancet Oncol.

[4] Van Rossum PSN, Fried DV, Zhang L (2016). The incremental value of subjective and quantitative assessment of 18F-FDG PET for the prediction of pathologic complete response to preoperative chemoradiotherapy in esophageal cancer. J Nucl Med.

[5] Piazzese C, Foley K, Whybra P (2019). Discovery of stable and prognostic CT-based radiomic features independent of contrast administration and dimensionality in oesophageal cancer. PLoS One.

[6] Beukinga RJ, Hulshoff JB, Mul VEM (2018). Prediction of response to neoadjuvant chemotherapy and radiation therapy with baseline and restaging 18F-FDG PET imaging biomarkers in patients with esophageal cancer. Radiology.

[7] O’Shea RJ, Tsoka S, Cook GJR, Goh V (2021). Sparse regression in cancer genomics: comparing variable selection and predictions in real world data. Cancer Inform.

[8] Wasserman L, Roeder K (2009). High-dimensional variable selection. Ann Stat.

[9] Park JE, Park SY, Kim HJ, Kim HS (2019). Reproducibility and generalizability in radiomics modeling: possible strategies in radiologic and statistical perspectives. Korean J Radiol.

[10] Foley KG, Hills RK, Berthon B (2018). Development and validation of a prognostic model incorporating texture analysis derived from standardised segmentation of PET in patients with oesophageal cancer. Eur Radiol.

[11] Traverso A, Wee L, Dekker A, Gillies R (2018). Repeatability and reproducibility of radiomic features: a systematic review. Int J Radiat Oncol Biol Phys.

[12] O’Shea RJ, Sharkey AR, Cook GJR, Goh V (2021). Systematic review of research design and reporting of imaging studies applying convolutional neural networks for radiological cancer diagnosis. Eur Radiol.

[13] Demircioğlu A (2021). Measuring the bias of incorrect application of feature selection when using cross-validation in radiomics. Insights Imaging.

[14] Chalkidou A, O’Doherty MJ, Marsden PK (2015). False discovery rates in PET and CT studies with texture features: a systematic review. PLoS One.

[15] Lambin P, Leijenaar RTH, Deist TM (2017). Radiomics: the bridge between medical imaging and personalized medicine. Nat Rev Clin Oncol.

[16] van Rossum PSN, Xu C, Fried DV (2016). The emerging field of radiomics in esophageal cancer: current evidence and future potential. Transl Cancer Res.

[17] Concato J, Peduzzi P, Holford TR, Feinstein AR (1995). Importance of events per independent variable in proportional hazards analysis I. Background, goals, and general strategy. J Clin Epidemiol.

[18] Peduzzi P, Concato J, Feinstein AR, Holford TR (1995). Importance of events per independent variable in proportional hazards regression analysis II. Accuracy and precision of regression estimates. J Clin Epidemiol.

[19] Collins GS, Reitsma JB, Altman DG, Moons KGM (2015). Transparent reporting of a multivariable prediction model for individual prognosis or diagnosis (TRIPOD): the TRIPOD statement. BMJ.

[20] Zwanenburg A, Vallières M, Abdalah MA (2020). The image biomarker standardization initiative: standardized quantitative radiomics for high-throughput image-based phenotyping. Radiology.

[21] Wickham H (2016). ggplot2: elegant graphics for data analysis.

[22] R Core Team (2021). R: a language and environment for statistical computing.

[23] RStudio Team (2021). RStudio: integrated development for R.

[24] Page MJ, McKenzie JE, Bossuyt PM (2021). The PRISMA 2020 statement: an updated guideline for reporting systematic reviews. Syst Rev.

[25] Zhang C, Shi Z, Kalendralis P (2021). Prediction of lymph node metastases using pretreatment PET radiomics of the primary tumour in esophageal adenocarcinoma: an external validation study. Br J Radiol.

[26] Baiocco S, Sah BR, Mallia A (2019). Exploratory radiomic features from integrated 18 F-fluorodeoxyglucose positron emission tomography/magnetic resonance imaging are associated with contemporaneous metastases in oesophageal/gastroesophageal cancer. Eur J Nucl Med Mol Imaging.

[27] Beukinga RJ, Wang D, Karrenbeld A (2021). Addition of HER2 and CD44 to 18F-FDG PET-based clinico-radiomic models enhances prediction of neoadjuvant chemoradiotherapy response in esophageal cancer. Eur Radiol.

[28] Yip SSF, Coroller TP, Sanford NN (2016). Relationship between the temporal changes in positron-emission-tomography-imaging-based textural features and pathologic response and survival in esophageal cancer patients. Front Oncol.

[29] Simoni N, Rossi G, Benetti G (2020). 18F-FDG PET/CT metrics are correlated to the pathological response in esophageal cancer patients treated with induction chemotherapy followed by neoadjuvant chemo-radiotherapy. Front Oncol.

[30] Zhang YH, Herlin G, Rouvelas I (2019). Texture analysis of computed tomography data using morphologic and metabolic delineation of esophageal cancer—relation to tumor type and neoadjuvant therapy response. Dis Esophagus.

[31] Klaassen R, Larue RTHM, Mearadji B (2018). Feasibility of CT radiomics to predict treatment response of individual liver metastases in esophagogastric cancer patients. PLoS One.

[32] Wagner F, Hakami YA, Warnock G (2017). Comparison of contrast-enhanced CT and [18F]FDG PET/CT analysis using kurtosis and skewness in patients with primary colorectal cancer. Mol Imaging Biol.

[33] Altman DG, Royston P (2006). The cost of dichotomising continuous variables. Br Med J.

[34] Tomasello G, Petrelli F, Ghidini M (2017). Tumor regression grade and survival after neoadjuvant treatment in gastro-esophageal cancer: a meta-analysis of 17 published studies. Eur J Surg Oncol.

[35] Yip SSF, Coroller TP, Sanford NN (2016). Use of registration-based contour propagation in texture analysis for esophageal cancer pathologic response prediction. Phys Med Biol.

[36] Tixier F, Le Rest CC, Hatt M (2011). Intratumor heterogeneity characterized by textural features on baseline 18F-FDG PET images predicts response to concomitant radiochemotherapy in esophageal cancer. J Nucl Med.

[37] Yip C, Landau D, Kozarski R (2013). Primary esophageal cancer: heterogeneity as potential prognostic biomarker in patients treated with definitive chemotherapy and radiation therapy. Radiology.

[38] Hatt M, Tixier F, Cheze Le Rest C (2013). Robustness of intratumour 18F-FDG PET uptake heterogeneity quantification for therapy response prediction in oesophageal carcinoma. Eur J Nucl Med Mol Imaging.

[39] Tan S, Kligerman S, Chen W (2013). Spatial-temporal [18F]FDG-PET features for predicting pathologic response of esophageal cancer to neoadjuvant chemoradiation therapy. Int J Radiat Oncol Biol Phys.

[40] Larue RTHM, Van De Voorde L, van Timmeren JE (2017). 4DCT imaging to assess radiomics feature stability: an investigation for thoracic cancers. Radiother Oncol.

[41] Larue RTHM, Klaassen R, Jochems A (2018). Pre-treatment CT radiomics to predict 3-year overall survival following chemoradiotherapy of esophageal cancer. Acta Oncol.

[42] Her EJ, Haworth A, Reynolds HM (2020). Voxel-level biological optimisation of prostate IMRT using patient-specific tumour location and clonogen density derived from mpMRI. Radiat Oncol.

[43] Foley KG, Shi Z, Whybra P (2019). External validation of a prognostic model incorporating quantitative PET image features in oesophageal cancer. Radiother Oncol.

[44] Johnson WE, Li C, Rabinovic A (2007). Adjusting batch effects in microarray expression data using empirical Bayes methods. Biostatistics.

[45] Karahan Şen NP, Aksu A, Çapa Kaya G (2021). A different overview of staging PET/CT images in patients with esophageal cancer: the role of textural analysis with machine learning methods. Ann Nucl Med.

[46] Howe CJ, Cole SR, Lau B (2016). Selection bias due to loss to follow up in cohort studies. Epidemiology.

[47] Tariman JD, Doorenbos A, Schepp KG (2014). Information needs priorities in patients diagnosed with cancer: a systematic review. J Adv Pract Oncol.

[48] Whybra P, Parkinson C, Foley K (2019). Assessing radiomic feature robustness to interpolation in 18F-FDG PET imaging. Sci Rep.

[49] Parkinson C, Foley K, Whybra P (2018). Evaluation of prognostic models developed using standardised image features from different PET automated segmentation methods. EJNMMI Res.

[50] Hatt M, Majdoub M, Vallières M (2015). 18F-FDG PET uptake characterization through texture analysis: investigating the complementary nature of heterogeneity and functional tumor volume in a multi-cancer site patient cohort. J Nucl Med.

[51] Moons KGM, Kengne AP, Woodward M (2012). Risk prediction models: I. Development, internal validation, and assessing the incremental value of a new (bio)marker. Heart.

[52] Kao YS, Hsu Y (2021). A meta-analysis for using radiomics to predict complete pathological response in esophageal cancer patients receiving neoadjuvant chemoradiation. In Vivo.

[53] Park JE, Kim HS, Kim D (2020). A systematic review reporting quality of radiomics research in neuro-oncology: toward clinical utility and quality improvement using high-dimensional imaging features. BMC Cancer.

[54] Wang H, Yang F, Luo Z (2016). An experimental study of the intrinsic stability of random forest variable importance measures. BMC Bioinform.

